# Production of Chitooligosaccharides and Their Potential Applications in Medicine

**DOI:** 10.3390/md8051482

**Published:** 2010-04-27

**Authors:** Berit B. Aam, Ellinor B. Heggset, Anne Line Norberg, Morten Sørlie, Kjell M. Vårum, Vincent G. H. Eijsink

**Affiliations:** 1 Department of Chemistry, Biotechnology and Food Science, Norwegian University of Life Sciences, 1432 Ås, Norway; E-Mails: berit.aam@umb.no (B.B.A.); anne.line.norberg@umb.no (A.L.N.); morten.sorlie@umb.no (M.S.); 2 Norwegian Biopolymer Laboratory (NOBIPOL), Department of Biotechnology, Norwegian University of Science and Technology, 7491 Trondheim, Norway; E-Mails: ellinor.heggset@biotech.ntnu.no (E.B.H.); kjell.vaarum@biotech.ntnu.no (K.M.V.)

**Keywords:** chitooligosaccharide (CHOS), chitinase, chitosanase, chitosan, application

## Abstract

Chitooligosaccharides (CHOS) are homo- or heterooligomers of *N*-acetylglucosamine and *D*-glucosamine. CHOS can be produced using chitin or chitosan as a starting material, using enzymatic conversions, chemical methods or combinations thereof. Production of well-defined CHOS-mixtures, or even pure CHOS, is of great interest since these oligosaccharides are thought to have several interesting bioactivities. Understanding the mechanisms underlying these bioactivities is of major importance. However, so far in-depth knowledge on the mode-of-action of CHOS is scarce, one major reason being that most published studies are done with badly characterized heterogeneous mixtures of CHOS. Production of CHOS that are well-defined in terms of length, degree of *N*-acetylation, and sequence is not straightforward. Here we provide an overview of techniques that may be used to produce and characterize reasonably well-defined CHOS fractions. We also present possible medical applications of CHOS, including tumor growth inhibition and inhibition of T_H_2-induced inflammation in asthma, as well as use as a bone-strengthener in osteoporosis, a vector for gene delivery, an antibacterial agent, an antifungal agent, an anti-malaria agent, or a hemostatic agent in wound-dressings. By using well-defined CHOS-mixtures it will become possible to obtain a better understanding of the mechanisms underlying these bioactivities.

## 1. Introduction to Chitin, Chitosans and Chitooligosaccharides (CHOS)

Chitin is a linear polysaccharide consisting of *β*(1→4) linked *N*-acetyl-*D*-glucosamine (GlcNAc; **A**) residues. It is often considered the second most abundant polysaccharide in nature, after cellulose, and occurs mainly as a structural component in the cell walls of fungi and yeasts and in the exoskeletons of insects and arthropods (e.g., crabs, lobsters and shrimps). Chitin is insoluble in water and exists mainly in two crystalline polymorphic forms, α and β. α-Chitin consists of sheets of tightly packed alternating parallel and antiparallel chains [1] and is found in the exoskeleton of arthropods, in insects and in fungal and yeast cell walls. In β-chitin the chains are arranged in parallel [2]. β-chitin occurs less frequently in nature than α-chitin, but can be extracted from squid pens.

Chitosan can be prepared from chitin by partial deacetylation, and is a heteropolymer of GlcNAc and d-glucosamine (GlcN; **D**) residues. Unlike chitin, chitosan is soluble in dilute aqueous acid solutions. The name chitosan refers to a continuum of soluble polymeric chitin derivatives that can be described and classified according to the fraction of *N-*acetylated residues (F_A_) or degree of *N-*acetylation (DA), the degree of polymerization (DP) or the molecular weight (M_W_), the molecular weight distribution (PD, for PolyDispersity), and the pattern of *N-*acetylation (P_A_) or sequence. Chitosan exhibits a variety of interesting physicochemical and biological properties. This, in combination with its non-toxicity, biocompatibility and biodegradability, makes chitosan suitable for use in numerous applications in agriculture, cosmetics, water treatment and medicine [3–7].

Chitooligosaccharides (CHOS) are oligomers prepared from chitosan either chemically or enzymatically. Chitosan can be converted to CHOS by acid hydrolysis or by enzymatic hydrolysis with glycosyl hydrolases like chitinases or chitosanases. The F_A_, M_W_, PD and P_A_ of the resulting CHOS-mixture depend on the chitosan and the specificity of the enzyme used. As described below, product mixtures can be enriched for certain compounds by optimizing the chitosan-enzyme combination.

There is ample literature concerning the biological effects of chitosans, but relatively little is known about the effects of CHOS [6,8], and the mechanisms behind observed bioactivities are generally poorly understood. CHOS are primarily thought to interact with proteins that either act on chitin (e.g., chitinases) or that are supposed to bind to and detect chitin. When it comes to medicine, there are indications that CHOS may be developed as drugs against asthma [9–12], antibacterial agents [13], ingredients in wound-dressings [14,15] and vectors in gene-therapy [16,17]. Furthermore, according to the literature, CHOS may reduce metastasis and tumor-growth in cancer [18–20], increase bone-strength in osteoporosis [21,22] and could be used to inhibit chitinases in *Plasmodium* parasites and thereby prevent malaria [23]. Several other potential effects of CHOS have been described, including immune modulatory effects [24], anti-fungal activities [25,26] and a lowering effect on serum glucose levels in diabetics [27]. In the context of this review, it should be noted that some of the biological effects reported for chitosan in fact may be due to CHOS, which emerge when chitosan is degraded by naturally occurring hydrolytic enzymes.

So far, most of the research on bioactivities of CHOS has been done with badly defined mixtures containing CHOS of various F_A_, M_W_, PD and P_A_. Moreover, the CHOS fractions appearing in the literature are derived from various sources of chitosan that are not always traceable and that have been characterized to only a limited extent. Clearly, when using complex mixtures of CHOS in bioassays, it is difficult to know which molecule/molecules are causing the effects. Furthermore, reproducibility can be an issue. All in all, while interesting biological activities have been observed, there is little information on the molecular properties that confer bioactivity to a particular CHOS.

In this review we provide an overview of some of the most promising applications of CHOS. Furthermore, we describe current knowledge on how well-defined mixtures of CHOS can be prepared and characterized. It is possible to prepare CHOS from chitosan by using different physical methods, like hydrothermal [28], microwave [29], ultrasonication [30] and gamma-rays [31], but these methods are not optimal for creating well-defined CHOS-mixtures and will not be dealt with in this review. Chemical methods using acid [32,33], H_2_O_2_ [34] or NaNO_2_ [35], can yield CHOS and one of these methods is described briefly below. We will, however, focus our review on the enzymatic production of CHOS, and on further separation and purification methods for producing well-defined mixtures.

## 2. Chitosan, the Starting Material for CHOS Production

It is possible to isolate chitosan directly from the cell walls of certain fungi, but commercially available chitosans are usually prepared from chitin in a heterogeneous deacetylation process. Chitosans will in this paper be defined as proposed by Roberts [36], *i.e.*, according to their solubility at acidic pH-values. This distinguishes clearly between chitins and chitosans, although some controversy may exist when discussing chitin oligomers (*N*-acetyl-CHOS) and chitosan oligomers (CHOS). To avoid this problem, we restrict the terms chitin and chitosan to polymers with more than 100 sugar units.

Chitosans can be prepared from chitin by two fundamentally different methods, *i.e.*, homogeneous [37] and heterogeneous deacetylation. In the homogeneous deacetylation process, the chitin is dissolved in an alkali solution during the deacetylation process (at low temperature and using excessive stirring). In the heterogeneous deacetylation process, the chitin is kept insoluble in a hot alkali solution, meaning that this is a two-phase process. Chitosans with F_A_ varying from 0 to about 65% can be prepared by homogeneous deacetylation of chitin [37]. There is little controversy regarding the distribution (P_A_) of the two monomers in these chitosans, which have been found to have a random distribution of the GlcNAc and GlcN units, *i.e.*, according to Bernoullian distribution [38–40]. There is, however, more controversy about the distribution of sugar units in chitosan prepared by heterogeneous deacetylation procedures. Weinhold *et al.* [41] studied the *N-*acetylation patterns of 32 different chitosans of various F_A_, using a previously described NMR-method [39], most of them prepared by heterogenous deacetylation, and found a close to random distributions for all samples. Although this recent study indicates that the P_A_ in chitosans prepared by heterogenous deacetylation is more random than previously assumed, it should be kept in mind that the NMR-method is only capable of determining an average distribution, meaning that certain block patterns may in fact exist.

The annual production of chitin in nature has been estimated to approximately 10^10^–10^11^ tons per year [42]. The amounts of chitin, chitosan and their derivatives that are used/produced in industrial processes [43] have been estimated to be about 30 000 metric tons for chitin and about 10,000 metric tons for chitosan in 2007 [44]. Most of the chitin is used as raw material for the production of the monosaccharide GlcN, which is the number one dietary supplement in the USA, used for pain relief of osteoarthritis [43].

## 3. Enzymes Acting on Chitin and Chitosan

### 3.1. Chitinases and chitosanases

**Figure 1:**
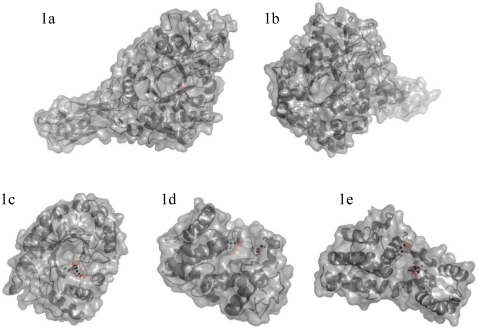
Structures of the enzymes discussed in detail in this review. Figure 1a and 1b show, respectively, ChiA and ChiB from *Serratia marcescens*. Figure 1c shows hevamine, a plant family 18 chitinase whose structure is thought to resemble the (unknown) structure of the catalytic domain of ChiC from *Serratia marcescens*. Figure 1d shows ChiG from *Streptomyces coelicolor* A3(2). Figure 1e shows CsnN174, a family 46 chitosanase from *Streptomyces* sp. N174, which, judged from sequence similarity, is highly similar to Csn88 from *Streptomyces coelicolor* A3(2). The side chains of the catalytic acid and of the catalytic base/nucleophile are shown.

**Figure 2:**
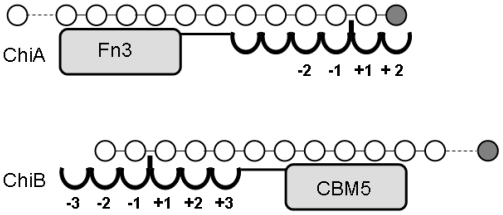
Schematic drawing of subsites, chitin binding domains and proposed orientation of polymeric substrates in ChiA and ChiB. Fn3, Fibronectin type 3 domain (substrate-binding); CBM5, chitin binding module. Dotted lines indicate that the polymer substrates are much longer than shown in the figure. Reducing end sugars are shown in grey. Figure and legend are from Horn *et al.* [56], and is reproduced with permission from Wiley-Blackwell.

Enzymatic depolymerization of chitin and chitosan involves chitinases and chitosanases, respectively (Figure 1). These enzymes hydrolyze the glycoside bonds between the sugars and are thus glycoside hydrolases (GH). Such GH are classified in the Carbohydrate-Active enZYmes database (CAZy) [45–48] that provides a continuously updated list of the GH families and, since a few years, also other families of carbohydrate-active enzymes such as glycosyl transferases and carbohydrate esterases. The CAZy classification is based on the amino acid sequence, which gives very useful information since sequence and structure, and hence mechanism, are related. It should be noted that the CAZy system does not take substrate and product activities into account *a priori*. The same applies to enzyme properties such as exo- *versus* endo-binding, processivity, and the presence of additional modules that may improve substrate-binding. All these properties are not taken into account either. The CAZy classification is purely based on amino acid sequence similarities and, indeed, several GH classes contain enzymes acting on a variety of substrates. Many GH enzymes have one or more carbohydrate binding domains in addition to their catalytic domain (Figures 1 and 2). These are referred to as carbohydrate-binding modules (CBMs) and are also classified in the CAZy database.

Chitinases occur in families GH18 and GH19 and both these classes almost exclusively contain these enzymes. Chitinases have the unique ability to hydrolyze **A**-**A** bonds and this property discriminates these enzymes from chitosanases. However, as described below, chitinases are perfectly capable of hydrolyzing chitosan, albeit to different extents. Chitinases do not hydrolyze **D**-**D** bonds.

Enzymes with chitosanase activity have been found in GH families 5, 7, 8, 46, 75 and 80. GH7 is a cellulase family and in a very few cases chitosanase activity has been detected as a side activity of these enzymes. GH5 contains a variety of enzymatic activities, including chitosanases, cellulases, licheninases, mannanase and xylanases. Again, chitosanase activity has been detected in only a very few cases, and the activity seems to be a side activity of cellulases. In GH8, enzymes annotated as chitosanases occur more frequently (next to e.g., cellulases and xylanases), and this family seems to contain a few true chitosanases [49]. The other three families, GH46, GH75 and GH80, exclusively contain chitosanases. Families GH75 and GH80 have only a few members and there is no structural and only very little functional information available for these enzymes. The best studied chitosanases, by far, are those belonging to family 46 [50–52] (Figure 1).

Based on their substrate specificity towards chitosan, chitosanases have been classified into subclasses I, II and III [53]. Chitosanases in subclass I can hydrolyze **A**-**D** and **D**-**D** linkages, subclass II enzymes can hydrolyze **D**-**D** linkages only, whereas subclass III enzymes can hydrolyze **D**-**A** and **D**-**D** linkages. Subclass I enzymes have been found in both families 46 and 75 [54,55]. In family 46, subclass III enzymes have also been found [52].

### 3.2. Catalytic mechanism

The hydrolysis of the glycosidic linkage is a nucleophilic substitution at the anomeric carbon, and can lead to either retention or inversion of the anomeric configuration [57,58]. Both hydrolysis reactions take place through general acid catalysis, and require a pair of carboxylic acids at the enzyme’s active site. One carboxylic acid is acting as a proton donor, facilitating leaving group departure, and the other acts as a base (inverting mechanism) or as a nucleophile (retaining mechanism). In both mechanisms, the position of the proton donor is within hydrogen-bonding distance of the glycosidic oxygen. The inverting mechanism (also called the single displacement mechanism) is a “one-step” reaction, where the protonation of the glycosidic oxygen occurs simultaneously with a nucleophilic attack on the anomeric carbon by an activated water molecule. This water molecule is located between a carboxylic group and the anomeric carbon and it is activated by the carboxylic group that acts as a base. Since the water molecule approaches the anomeric carbon from the side of the catalytic base, this mechanism leads to inversion of the anomeric configuration. Chitosanases belonging to families GH46, GH75 and GH80 and family 19 chitinases use the inverting mechanism [45,47,49,54,59–61].

The retaining mechanism (also referred to as the double displacement mechanism) is a two-step reaction, were the first step involves the protonation of the glycosidic oxygen (by the catalytic acid) and a congruent nucleophilic attack on the anomeric carbon atom by the nucleophile (the second carboxylic acid). This attack leads to breakage of the glycosidic linkage and the formation of a covalent linkage between the anomeric carbon and the catalytic nucleophile [62]. Subsequently, this intermediate is hydrolyzed by a water molecule that approaches the anomeric carbon from a position close to that of the original glycosidic oxygen, leading to retention of the anomeric carbon configuration.

Family 18 chitinases use a special variant of the double displacement mechanism, referred to as the substrate-assisted double displacement mechanism. Here, the carbonyl oxygen atom from the *N*-acetylgroup of the sugar bound in subsite -1 act as the nucleophile, leading to formation of an oxazolinium ion intermediate. Because of this involvement of the *N*-acetylgroup in catalysis, productive substrate-binding of chitosan and chitosan oligomers to family 18 chitinases requires that a GlcNAc is bound in the -1 subsite [63–67].

Chitinases and chitosanases can have endo- or exo-activity, where both the endo- and exo-mode of action can be combined with processivity. Processive enzymes will not release the substrate after one cleavage, but remain associated with the substrate so that a new cleavage can take place as the polymer substrate slides through the substrate-binding cleft (Figure 2). Processivity is difficult to analyze when degrading insoluble substrates such as chitin, but can be studied when using chitosan as substrate [56,64,68,69]. Whereas processivity generally is considered to be favorable for the hydrolysis of crystalline substrates, processivity has been shown to reduce enzyme efficiency towards soluble and more accessible polymeric substrates such as chitosan [69,70]. Thus, for the industrial production of CHOS, the use of non-processive enzyme variants may be beneficial in some cases.

### 3.3. Human chitinases

Even though chitin and chitin synthases have not been found in humans, we produce two family 18 chitinases with chitinolytic activity [71]. In addition, two highly homologous proteins named chi-lectins and a homologous protein called oviductin are expressed. These proteins lack enzymatic activity while having retained the typical features of family 18 enzymes, including carbohydrate binding to the active site [71].

One of the human chitinases, called chitotriosidase (HCHT), is synthesized and secreted as a 50-kDa two-domain protein in human macrophages [72]. A significant portion of produced enzyme is routed to lysosomes and processed into a 39-kDa isoform, lacking the C-terminal chitin-binding domain [73]. HCHT was first discovered as a marker for Gaucher disease [72], but has later been associated with several diseases like malaria [74], fungal infections such as candidosis [75], sarcoidosis [76,77], cardiovascular risk [78], coronary artery disease [79], primary prostate cancer and benign prostatic hyperplasia [80], nonalcoholic steatohepatitis [81], multiple sclerosis [82], and Niemann-Pick disease [83]. The other chitinase, acidic mammalian chitinase (AMCase), is also synthesized as a two-domain 50 kDa protein containing a 39 kDa N-terminal catalytic domain and a C-terminal chitin-binding domain. AMCase is found in the stomach [84], in tears [85], sinus mucosa [86], and lungs [12,84]. Chitinases play important roles in the antiparasite responses of lower life forms [87–89]. Both HCHT and AMCase are believed to play similar roles in the human immune defense system, being a part of antiparasitic responses [10,87,90].

The two chi-lectins are associated with numerous diseases. YKL-40 (alternatively called HCgp39 or CHI3L1) is observed in elevated levels for patients with severe asthma [91], cardiovascular disease and diabetes [92], cancer [93], peritoneal endometriosis [94], morbid obesity [95], osteoarthritis [96], and liver fibrosis [97]. The other chi-lectin YKL-39 (or CHI3L2) has been observed up-regulated in osteoarthritic chondrocytes [98] and osteoarthritic cartilage [99]. The loss of hydrolytic activity in these chi-lectins is due to replacement of the catalytic acid (E) and the adjacent aspartic acid (D) in the conserved DxxDxDxE motif with Ala and Leu or Ser and Ile for YKL-40 and YKL-39, respectively. The chi-lectins have retained their ability to bind CHOS [100] and this may be a feature underlying some of the alleged biological effects of CHOS.

### 3.4. Inhibition of family 18 chitinases with CHOS

Family 18 chitinases are of particular interest, because they are abundant in nature, are crucial in the life cycles of numerous plague and pest organisms, and because they occur in humans themselves. Because of the substrate-assisted catalytic mechanism of GH18 enzymes, catalysis requires that a GlcNAc is bound to the -1 subsite. CHOS that preferably bind in such a way that a GlcN ends up in the crucial -1 subsite will act as an inhibitor. Sugar binding to the -1 subsite leads to an energetically unfavorable distortion [101] which involves the *N*-acetylgroup [66,67] and which may amount to an unfavorable Δ*G* as high as ~8 kcal/mol [102]. Although this has not yet been substantiated by experimental data, it seems plausible that binding of GlcN in the -1 subsite in fact could be energetically less unfavourable than binding of a GlcNAc. Thus, binding of a GlcN would be non-productive, but perhaps stronger than binding of a GlcNAc. This shows that the idea of developing partially deacetylated CHOS as inhibitors for family 18 chitinases is worth pursuing. Indeed, the validity of this idea has been substantiated by an early study by Peter and co-workers [103] as well as by more recent work [104,105].

For chitinase B of *Serratia marcescens*, the -2 subsite has a strong preference for an GlcNAc [67]. The oxygen atom in the acetamidogroup of the -2 sugar forms a bifurcated hydrogen bond with Trp ^403^ and Gln ^407^, whereas the methyl group packs tightly in an apolar environment provided by the side chains of Tyr ^292^ and Ile ^337^. Thus, the -2 subsite of this enzyme seems optimized for strong binding of an GlcNAc, which is not surprising taking into account that the positive effects of binding sugars in subsites adjacent to the -1 subsite is needed to “pull” the -1 sugar in its distorted conformation [106]. Indeed, one has observed non-productive binding by a **DADAA** oligomer bound from subsites -3 to +2 in chitinase B [104].

CHOS being based on the substrate, hold a tremendous advantage in being very specific inhibitors towards chitinases, and hence not likely to interfere with other enzymatic systems. Moreover, the binding strength of the CHOS based inhibitor, an important parameter, can be tuned simply by increasing the chain length of the CHOS or by coupling additional groups to the reducing end.

### 3.5. Lysozyme

In addition to its natural substrate, the glycosidic linkage of certain bacterial cell wall peptidoglycans, lysozyme may also hydrolyze chitin and chitosans [107]. In very early work, Amano and Ito [107] studied oligomers formed upon lysozyme degradation of an F_A_ = 0.32 chitosan, and identified the fully *N-*acetylated trimer and tetramer together with the partially *N*-acetylated oligomers **AAD**, **DAA**, **AAAD**, **ADAA** and **ADAD** among the oligomeric products. Later, Vårum *et al.* [108] studied lysozyme degradation of a highly *N-*acetylated chitosan (F_A_ = 0.65). In this study, NMR-spectroscopy of the degradation products was used to determine the identities of the newly formed reducing and non-reducing ends. This methodology, which has later been used to characterize chitinases (see below), provided insight into the cleavage specificities of the enzyme, *i.e.*, its preference for cleaving **A-A**, **A-D**, **D-A** and/or **D-D** linkages in chitosans. Some information on the identity of the nearest neighbors to the new reducing and non-reducing ends could also be obtained.

## 4. CHOS Production—Enzymatic Methods

So far, there are no robust enzymatic methods for the production of chitosan that could provide an alternative to the current chemical production methods. In principle, chitin deacetylases could be used to produce chitosan [109–113]. These enzymes hydrolyze the *N-*acetyl linkage and convert GlcNAc to GlcN. However, the insolubility and crystallinity of the chitin substrate forms a major hurdle for this approach. Chitin deacetylases could also be used to modify the *N-*acetylation pattern of CHOS, but this route has so far remained unexplored.

Although there are routes for chemical conversion of chitosan to CHOS [7] (see below), even CHOS with specific DP and P_A_, enzyme technology probably is the most promising approach. The specificity of chitosan-degrading enzymes has traditionally been studied by extensive enzymatic degradation of the polymer and subsequent isolation and characterization of the resulting oligomers. More recently, studies with chitinases have shown that the kinetics of the degradation reactions is such that product profiles change considerably during the hydrolysis reaction. Because the enzymes have very different binding affinities for different sequences on the substrate, reactions show multiphasic kinetics, and the product mixtures obtained at the end of each of these phases differ considerably. Another important issue is processivity; degradation processes may change during a reaction, from initial mainly processive hydrolysis of polymeric chains to non-processive hydrolysis of intermediate products as the polymeric material becomes exhausted. All in all, this means that the choice of the starting chitosan, the choice of the enzyme, and the choice of the processing time all affect the outcome of the enzymatic conversion process and that there are ample opportunities to manipulate this outcome [114]. This is illustrated by several studies on enzymatic degradation of chitosans [56,60,107,114–117], some of which are discussed in detail below. Structures of the enzymes discussed below are shown in Figure 1, whereas Table 1 shows some key properties.

### 4.1. Degradation of chitosan by family 18 chitinases

**Figure 3:**
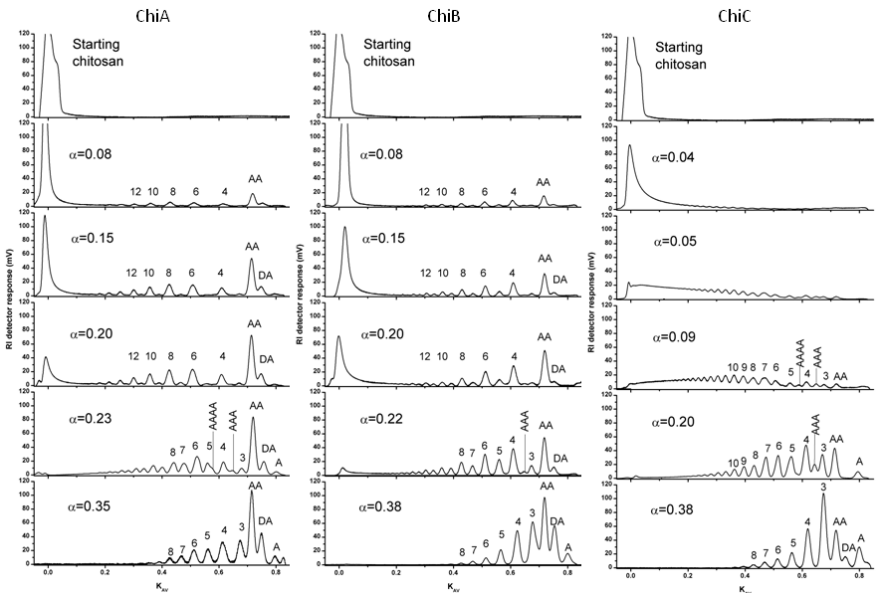
Degradation of chitosan (F_A_ 0.65) by ChiA, ChiB and ChiC from *Serratia marcescens*. The pictures show chromatograms from size-exclusion chromatography. The peaks are marked by numbers which indicate the lengths (DP) of the oligomers they contain, or, in the case of peaks containing only one known compound, by the sequence of the oligomer. The annotation of the peaks is based on the use of standard samples, as well as NMR analyses. The α-values denote the degree of scission [full conversion of the chitosan to dimers only (DP_n_ = 2) would give an α = 0.5 (α = 1/DP_n_)]. The lower panels represent the maximum obtainable α-values. Undegraded chitosan and fragments with a DP > 40 elute in the void volume of the column. The figure is from Horn *et al.* [56], and is reproduced with permission from Wiley-Blackwell. Additional product profiles at very low α for ChiA and ChiB that clearly reveal processivity have been published in Sikorski *et al.* [118].

The degradation of chitosan by the family 18 chitinases, ChiA, ChiB and ChiC, from *Serratia marcescens* has been studied in much detail [56,64,69,118]. Figure 3 shows the size-distribution of oligomers obtained upon degradation of a highly *N-*acetylated chitosan (F_A_ = 0.65) to various extents of degradation (α). For ChiA and ChiB, the product profiles obtained during the initial phase of the degradation show a dominance of even-numbered oligomers, which is indicative of processive action [56,64,68]. This product pattern is due to the fact that enzyme-ligand complexes where there is a GlcN bound to the -1 subsite is not productive (in the case of family 18 chitinases). If the enzyme is processive, the enzyme will slide by two sugar units at the time, until a productive complex is formed [the primary condition being that there is an GlcNAc bound in the -1 subsite (see Sørbotten *et al.* [64] and Eijsink *et al*. [68] for a more detailed discussion)]. Consequently, while the first product of an enzyme-substrate association may have any length, every subsequent product will be even-numbered. In the case of chitin, all these products would be dimers; in the case of chitosan, these products may be longer even-numbered oligomers. Later during the reaction, the dominance of even-numbered oligomers disappears because there are no longer substrate molecules left and the enzyme is primarily involved in rebinding and further cleavage of oligomers from the preceding processive phase (see Figure 3). For example, an oligomer such as **ADADAA** that could emerge during processive degradation by ChiB can be converted by this same enzyme to **ADA** and **DAA** upon rebinding in a mode that was not explored during the processive phase, where the substrate moves by two sugars at the time (see Horn *et al.* [56] and Eijsink *et al.* [68] for a more extensive discussion and explanation).

Figure 3 shows a totally different product pattern for ChiC that is characteristic for an endo-acting, non-processive enzyme. ChiC converts chitosan to a continuum of oligomers of different sizes and the polymer peak disappears early in the degradation reaction. Also, there is initially no accumulation of dimers or other even numbered oligomers. This all indicates that ChiC attacks the polymeric substrate chains in random positions, without processivity. Indeed, this “endo” activity could be confirmed by viscosity measurements during the hydrolysis reaction [118]. The contrast with ChiA and ChiB is perhaps best illustrated by the void peak, which disappears much more slowly in the case of processive enzymes. These latter enzymes perform many cuts per chain instead of a few cuts in every chain as in the case of ChiC.

**Figure 4:**
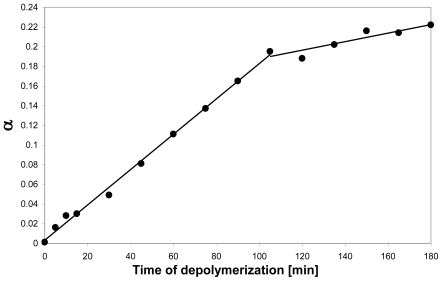
Time course of the degradation of a chitosan with F_A_ 0.65 by ChiB from *Serratia marcescens*. The graph shows the degree of scission (α) as a function of time; the biphasic kinetics is clearly visible. The slow phase continues until α reaches a value of about 0.37. Figure from Sørbotten *et al.* [64]. Reproduced with permission from Wiley-Blackwell.

Analysis of the sequences of the products (Table 2) showed considerable differences between the enzymes as well as differences over the time course of the degradation reactions. In all three enzymes, productive binding requires a GlcNAc in subsite -1, explaining why all products have a GlcNAc at their reducing ends. The enzymes did not have any detectable preferences for GlcNAc versus GlcN in the +1 subsite, and this is reflected in the oligomers having both *N-*acetylated and deacetylated non-reducing ends. All three chitinases showed a strong, but not absolute, preference for GlcNAc in subsite -2, meaning that oligomers preferentially have an *N-*acetylated unit next to the reducing end. The kinetics of the reaction with ChiB illustrate this (Figure 4; Table 2): in the initial rapid phase of the reaction almost all oligomeric producs have −**AA** at their reducing ends; during the second, much slower phase, oligomeric products ending at −**DA** appear to a larger extent [56, 64]. The preference for a GlcNAc in subsite -2 was strongest and in fact almost absolute for ChiC; all oligomeric products end with −**AA**, at any point during the reaction (Table 2).

**Figure 5:**
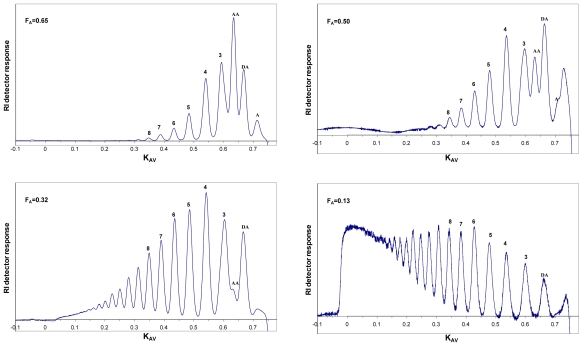
Size-distribution of oligomers after extended hydrolysis of various chitosans with ChiB from *Serratia marcescens*. The pictures show chromatograms revealing the size-distribution of oligomers obtained upon extended hydrolysis of chitosans with F_A_ of 0.65, 0.50, 0.32 and 0.13 to α-values (corresponding DP_n_-values in brackets) of 0.37 (2.7), 0.34 (2.9), 0.22 (4.5) and 0.11 (9.5), respectively. Figure from Sørbotten *et al*. [64]. Reproduced with permission from Wiley-Blackwell.

Figure 5 shows how the outcome in terms of the length distribution of products can be manipulated by varying the F_A_ of the chitosan. Obviously, since we are working with chitinases with clear preferences for GlcNAcs at certain positions in the substrate, the products get longer as the F_A_ goes down. It is quite remarkable that a chitinase such as ChiB works well on chitosans with F_A_ close to only 10%.

**Figure 6:**
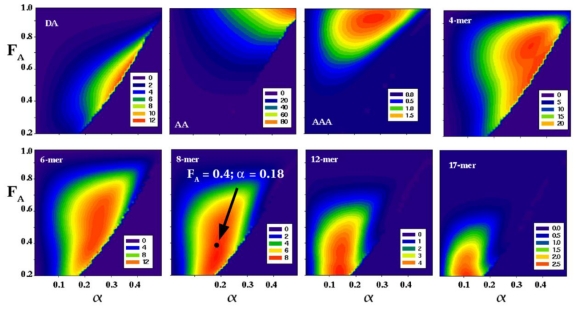
2D profiles showing the predicted outcome of chitosan hydrolysis with ChiB from *Serratia marcescens*. The X-axis shows the degree of scission, α, and the Y-axis shows the F_A_ of the starting chitosan. The predicted amount of a particular product at specific α - F_A_ combinations is indicated by color (the amounts of oligomers are expressed as % of the total mass of the polymer in the hydrolysis reaction and color coded as defined in the inserts). These profiles allow for selection of optimal reaction and substrate parameters for efficient production of oligomers with desired lengths. For example, high yields of octamer could be obtained if chitosan with F_A_ 0.4 is hydrolyzed to α = 0.18 (the arrow indicates the maximum level of octamers). For example, for the octamer, at maximum yield conditions, approximately 8% of the polymer is expected to be converted to octamers. Figure taken from Sikorski *et al.* [114], and reproduced with permission from Wiley-Blackwell.

Sikorski *et al.* [114,118] have produced a model for the degradation of different chitosans with ChiB, which is capable of accurately predicting the outcome of hydrolysis reactions in terms of the length distributions of the products at varying α. This model can be used to predict how reactions need to be set up in order to maximize the production of CHOS of certain lengths (Figure 6).

### 4.2. Degradation of chitosan by family 19 chitinases

**Figure 7:**
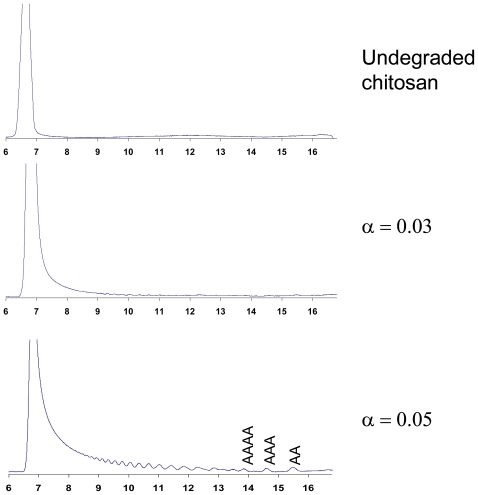

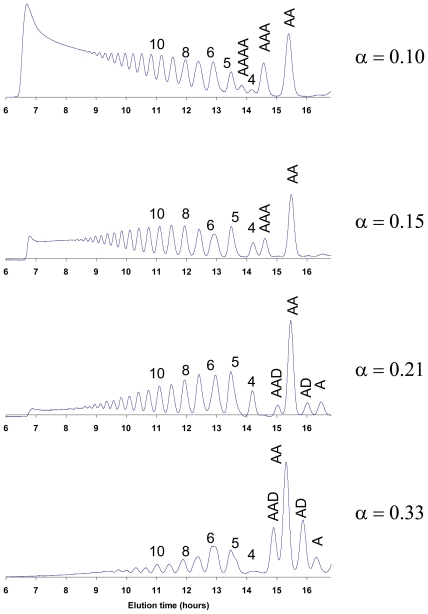
Size-distribution of oligomers emerging during hydrolysis of chitosan with F_A_ 0.64 by ChiG from *Streptomyces coelicolor* A3(2). See legend to Figure 3 for further explanation. Figure from Heggset *et al*. [115]. Reproduced with permission from American Chemical Society.

ChiG, a bacterial family 19 chitinase from *Streptomyces coelicolor* A3(2), produces quite different CHOS as compared to the family 18 chitinases, reflecting the very different binding preferences in the active sites of the enzymes [115]. Since ChiG, which operates according to a non-processive endo-mode of action (Figure 7), uses the inverting mechanism, there is no absolute requirement for GlcNAc in subsite -1. This means that the reducing ends of the oligomeric products could be both *N-*acetylated and deacetylated, as was indeed observed. The non-reducing ends of the oligomers were found to be exclusively *N-*acetylated, and the sugar units in the neighboring position to reducing ends were also found to be exclusively *N-*acetylated. Thus, ChiG has an absolute preference for a GlcNAc in subsites -2 and +1. The enzyme also has considerable preference for GlcNAc in -1 and kinetics were clearly biphasic: In the first fast phase cleavage occurred in **A-A**^↓^**A** sequences, whereas **A-D**^↓^**A** sequences were cleaved in the slower second phase [115]. So, while the chromatograms of Figure 7 may look somewhat similar to the chromatogram for the family 18 enzyme ChiC in Figure 3, the sequences of the produced oligomers show considerable differences, which may affect bioactivity. More generally, it is clear that ChiG will yield oligomers of different P_A_ as compared to those obtained by ChiA, ChiB and ChiC. For example, degrading a chitosan with F_A_ = 0.65 with ChiB and ChiG gives the trimers **DAA** and **AAD**, respectively [64,115].

**Figure 8:**
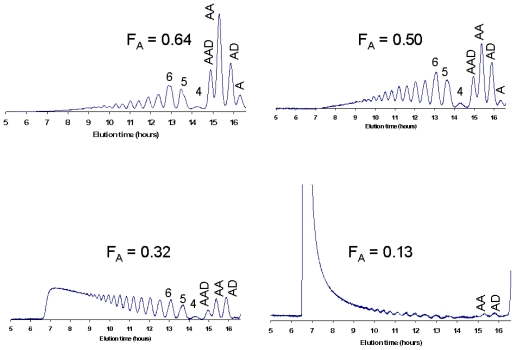
Size-distribution of oligomers after extended hydrolysis of various chitosans with ChiG from *Streptomyces coelicolor* A3(2). The chitosans with F_A_ of 0.13, 0.32, 0.50, and 0.64 were degraded to maximum α-values of 0.04, 0.12, 0.23, and 0.33, respectively. Peaks are labeled as in Figure 3. Figure from Heggset *et al*. [115]. Reproduced with permission from American Chemical Society.

Figure 8 shows that varying the F_A_ of the chitosan had a major effect on the size distribution of the products and the extent of cleavage. Interestingly, ChiG efficiency is much more sensitive for deacetylation than the efficiency of ChiB (compare Figure 8 with Figure 5).

Other studies on the degradation of chitosan with family 19 chitinases confirm the findings for ChiG. In one study, the family 19 chitinase ChiC from *Streptomyces griseus* HUT 6037 was used to (extensively) degrade a chitosan with a degree of *N-*acetylation of 47% and the products were isolated and characterized [116]. The identities of the isolated CHOS were **AD**, **AAD**, **ADAD**, **ADDAA** and **AADDAA**, and it was concluded that this enzyme has an absolute specificity for *N-*acetylated units in the -2 and +1 subsites, in agreement with the results for ChiG from *Streptomyces coelicolor*. A family 19 chitinase from rice [117] has been found to operate according to a non-processive endo-mode of action, with strong preferences for *N-*acetylated units in subsites -2 and +1, and with less strong preference for *N-*acetylated units in the -1 subsite.

### 4.3. Degradation of chitosan by family 46 chitosanases

The GH 46 family of chitosanases comprises enzymes classified as subclass I (cleaving **A-D** and **D-D** linkages) [60] as well as subclass III (cleaving **D-A** and **D-D** linkages) [120]. In an early study, a chitosan with F_A_ = 0.25–0.35 was extensively degraded with CsnN174 from *Streptomyces* sp. N174 and the oligomeric products were isolated and characterized. In addition to the fully deacetylated oligomers **D**, **DD** and **DDD**, several hetero-oligomers were identified (the dimer **DA**, the trimers **DDA** and **DAA**, the tetramers **DDAA** and **DAAA** and the pentamer **DDAAA**) [60]. These results suggest that CsnN174 has a high specificity for a GlcN in the +1 subsite (new non-reducing ends), whereas both GlcNAc and GlcN seem to be acceptable in the -1 subsite (new reducing ends). Further insight into the properties of CsnN174 would require studies of oligomer production over time, as well as studies using chitosans of varying F_A_.

Recent work in our own laboratories on a family 46 chitosanase, Csn88, from *Streptomyces coelicolor* has shown that this enzyme is capable of degrading chitosans with varying F_A_, producing oligomers with both *N-*acetylated and deacetylated reducing ends [121]. ^1^H NMR spectroscopy analysis showed that when the new reducing ends were *N-*acetylated, the sugar binding in the neighbouring position (*i.e.*, binding in subsite -2) always was deacetylated. However, in oligomers with deacetylated sugars at the new reducing end, both *N-*acetylated and deacetylated sugar occurred in the neighbouring position (revealed by mass spectrometry). The identity of the new non-reducing ends was studied using ^13^C NMR spectroscopy and this analysis showed that initial products exclusively had deacetylated non-reducing ends, whereas *N-*acetylated non-reducing ends appeared later during the reaction. All in all, these preliminary data indicate that Csn88 can cleave **D-A**, **A-D** and **D-D** linkages. As expected maximum α values were high, for example 0.59 for a highly deacetylated chitosan (F_A_ 0.008) and 0.44 for a F_A_ 0.32 chitosan.

### 4.4. Degradation of chitosan by unspecific enzymes

Several authors have employed unspecific enzymes such as papaine and cellulases to degrade chitosans (e.g., [122–124]). Since the enzyme preparations used tend to be rather crude and derived from sources (fungi, plants) that are known to produce chitinolytic enzymes, there remains some doubt concerning which enzymes actually catalyze the hydrolysis reactions. However, for the practical purpose of producing CHOS, the use of (cheap) unspecific enzymes may be quite useful.

## 5. CHOS Production—Chemical Methods

### 5.1. Acid hydrolysis of chitosan

Of chemical methods for hydrolysis of chitosan [32–35], acid hydrolysis is probably the best known. Early studies on acid hydrolysis of chitosans had shown that it is possible to convert fully deacetylated chitosan to CHOS in concentrated hydrochloric acid [32]. In later studies [33], using a variety of chitosans, the acid-catalyzed degradation rates of chitosans were shown to depend on F_A_, and the initial degradation rate constant was found to increase in direct proportion to F_A_. Acid hydrolysis was found to be highly specific to cleavage of **A-A** and **A-D** glycosidic linkages, which were hydrolyzed with two to three orders of magnitude higher rates than the **D-D** and **D-A** linkages. This preference is probably due to a combination of two factors: (1) the presence of a positively charged amino group (as in GlcN) close to the glycosidic linkage is inhibitory, and (2) the presence of an acetamidogroup (as in GlcNAc) close to the glycosidic linkage may yield some degree of substrateassistance to the hydrolytic mechanism. In the same study it was shown that the rate of deacetylation was less than one-tenth of the rate of depolymerization in concentrated acid, whereas the two rates were found to be equal in dilute acid. It was suggested that this is due to these two processes having different reaction mechanisms with different rate-limiting steps [33].

### 5.2. Chemical synthesis of CHOS

Chemical synthesis of CHOS requires multiple protection and deprotection steps, and is today not a routine procedure. Chemical synthesis of CHOS gives rise to pure compounds, but most methods existing today are time consuming and require extensive use of organic solvents and/or are not capable of producing anything else than homo-oligomers. There are in fact only a few examples of chemically synthesized CHOS in the literature. Kuyama *et al*. [125] performed synthesis of fully deacetylated chitosan dodecamers starting with glucosamine monomers using phthalimido as the amino protective group. Aly *et al*. [126] reported a method for synthesis of fully *N-*acetylated CHOS from GlcN monomers using dimethylmaleoyl as an amino protective group for synthesis of chitotetraose and chitohexaose. Removal of the amino protective group and *N*-acetylation was performed in a one-pot reaction to give the desired products [127]. In principle it would be possible to combine the use of these two described protection methods to synthesize partly deacetylated CHOS, but this has so far not been reported (to our knowledge).

Trombotto *et al*. [128] have reported a method for chemical preparation of partly deacetylated CHOS from fully deacetylated high molecular weight chitosan. The starting chitosan was partially depolymerized using HCl to produce fully deacetylated oligomers that were fractionated by selective precipitation and ultrafiltration to yield a mixture of DP 2–DP 12. The oligomers were then partly *N-*acetylated using stochiometric amounts of acetic anhydride to reach the decided F_A_. In this way, CHOS fractions of DP between 2 and 12 were successfully prepared. The drawback of this method, as for the enzymatic preparation of CHOS, is the heterogeneity of the prepared CHOS.

In an early study, Letzel *et al.* [103] used a similar approach: chitosan with F_A_ 0.02 was hydrolyzed with HCl, oligomers were separated by DP using gel permeation chromatography and the resulting CHOS fractions were *N-*acetylated using substoichiometric amounts of acetic anhydride to control the F_A_. Interestingly, some of the fractions produced in this study inhibited the family 18 chitinase ChiB from *Serratia marcescens*.

In principle, chemoenzymatic synthesis provides opportunities to produce pure CHOS of defined DP, F_A_ and P_A_ without the use of extensive protection of the functional groups at the sugar unit. So far this has been done by allowing an oxazoline, imitating the intermediate of chitin hydrolysis, to act as a glycosyl donor in an enzyme-catalyzed reaction where another GlcN/GlcNAc unit acts as a glycosyl acceptor [129]. By using oxazoline oligomeric building blocks of specific DP, F_A_, and P_A_, longer specific CHOS can be made using this approach. The main disadvantage using the chemo-enzymatic approach is that the product is necessarily also a substrate for the enzyme, which can result in poor yields. To avoid this problem, modified enzymes with reduced hydrolytic activity may be employed. The enzyme modifications would need to be aimed at reducing hydrolytic power, while increasing binding strength for the glycosyl donor in the glycon subsites [130].

## 6. Purification and Characterization of CHOS

CHOS produced enzymatically or chemically normally consist of a mixture of oligomers differing in DP, F_A_ and P_A_. Several techniques for separation and purification of CHOS have been reported, like gelfiltration [64], ultrafiltration [131], and ion exchange [132] and metal affinity [133] chromatography. Often, such techniques need to be applied in combination to obtain homogeneous CHOS fractions. Despite some successful studies, the production of pure CHOS fractions is generally a time consuming and challenging task.

Preparative separation of CHOS is most commonly based on size, through size exclusion chromatography (SEC). Recently, good methods for the separation of oligomers up to DP 40 (individual oligomers up to DP 20) have been described [64], as illustrated by Figure 3, 5, 7 and 8. The SEC system used for producing the data displayed in this review is based on Superdex^TM^ 30 (GE Healthcare) columns that are coupled in series. The oligomers are detected using an online refractive index detector. This system allows separation of CHOS with similar DP values ranging from approximately DP 2 to DP 20, independently of F_A_ and P_A_ [64].

Further separation of CHOS can be achieved using cation-exchange chromatography, because protonated amino groups on the deacetylated sugars interact with the ion-exchange material. With this method CHOS of identical DP will be separated based on the number of deacetylated units [132]. A further partial separation of isobaric CHOS (identical F_A_, different P_A_) may be achieved using strong cation exchange chromatography. Although, the latter separations are promising and useful, baseline separation of isobaric CHOS has so far not been achieved [132]. In an alternative strategy, metal affinity chromatography has been successfully used for separation of shorter CHOS. CHOS have a strong affinity for Cu^2+^, and using copper as a chelating agent gives separation up to 90% of fully deacetylated CHOS of DP 3 and higher [133]. This has not been reported for *N-*acetylated mixtures of CHOS.

In order to characterize CHOS in terms of DP, F_A_ and P_A_, several techniques have been applied, primarily nuclear magnetic resonance (NMR) and mass spectrometry. Using NMR, it is possible to determine F_A_ in a chitosan or CHOS sample and to (partially) identify the P_A_ in shorter CHOS depending on the complexity of the oligomer mixture. Resonances detected using NMR reveal that the H-1 resonance of a reducing unit is sensitive to its nearest neighbor, making it possible to (partially) determine the P_A_ of an oligomer [64]. In addition, the P_A_ of dimers and trimers can be determined using NMR. The identity of the non-reducing end unit of an oligomer can be determined using ^13^C NMR, which in some instances also may reveal the identity of its nearest neighbor [108].

Modern mass spectrometry provides excellent tools for the identification of the DP and F_A_ of CHOS [104,134]. In 1997 Okafo *et al.* [135] reported a reductive amination of CHOS using 2-aminoacridone (AMAC), which is useful for tagging of the reducing end. Building on this labeling technique, Bahrke *et al.* [134] developed a method for sequencing of CHOS up to DP 12 using reducing end derivatization with AMAC. Starting with CHOS fractions of homogeneous DP they used matrix-assisted laser desorption ionization (MALDI) time-of-flight (TOF) postsource decay (PSD) mass spectrometry (MS) for sequence determination. Reducing end derivatization of CHOS using AMAC favors formation of Y-type ions, meaning that sugars are only cleaved after the oxygen in the *β*(1→4) linkage from the reducing end. Consequently, interpretation of the resulting mass spectra is quite straightforward. It should be noted though that the method has limitations when applied to mixtures.

In a later study from the same group [132], a second method for reductive amination, using 3-(acetylamino)-6-aminoacridine, was adopted. Combined detection and fragmentation of isobaric CHOS using MALDI iontrap MS^n^ was reported. The described technique makes it possible to simultaneously determine sequence and quantity of CHOS of identical DP but different F_A_ and P_A_ in an isobaric CHOS mixture.

## 7. Applications of CHOS

Literature contains numerous papers reporting a remarkably wide range of biological activities of CHOS. As discussed above, the molecular mechanisms behind these bioactivities are mostly unknown and so is the exact nature of the bioactive component. Many activities have been reported only once or twice, providing insufficient basis to make general conclusions about the applicability of CHOS. Below, we discuss a selection of studies that report such bioactivities, with focus on studies that contain some discussion of the molecular mechanisms involved.

### 7.1. Tumor growth inhibition

It has been known since the 1970s that CHOS have anti-tumor effects [18], and there is also evidence for positive effects of CHOS in reducing metastasis from tumors [19,20]. It was first suggested that the anti-tumor activity was due to the cationic properties of CHOS [18]. Later, M_W_ also was proposed to play a major role [136]. Maeda and Kimura [137] found that CHOS enhanced the natural killer activity in intestinal intraepitelial lymphocytes at the same time as they reduced tumor growth in mice, and suggested that this CHOS-activation of intestinal immune functions could be useful in treating tumors.

There are indications that apoptosis is involved in the tumor-reducing effects of CHOS. Harish Prashanth and Tharanathan [138] discovered that DNA from Ehrlich ascites tumor cells was fragmented after incubation with CHOS, an indication of apoptosis. CHOS have also been shown to induce apoptosis of human hepatocellular carcinoma cell via upregulation of the pro-apoptotic protein Bax [139].

In recent years, the hypothesis that the anti-tumor effects of CHOS are related to their inhibitory effects on angiogenesis has received some attention [138,140,141]. Angiogenesis is the formation of new capillary blood vessels from already existing blood vessels. This process is important for tumor formation, since tumor growth and metastasis require angiogenesis when the tumor reaches a certain size. Xiong *et al.* [142] compared effects of dimers to hexamers of fully deacetylated CHOS on angiogenesis and found that the hexamers were the most effective inhibitors, whereas Wang *et al.* [140] showed that *N-*acetylated CHOS were more effective in preventing angiogenesis than fully deacetylated CHOS, both *in vitro* and *in vivo*.

### 7.2. Asthma

AMCase is induced during T_H_2 inflammation through an interleukin (IL)-13 dependent mechanism and has been demonstrated to be heavily over-expressed in human asthmatic tissue [10,12]. Inhibition of the AMCase with the well known chitinase inhibitor allosamidin reduced the inflammation [12]. The fact that chitinases are a factor in host antiparasite responses and in asthmatic T_H_2 inflammation led to the hypothesis that asthma may be a parasite-independent antiparasite response [10], which again suggests that inhibition of AMCase is a potential target for asthma therapy [9–12]. It has been shown that partially deacetylated CHOS can function as inhibitors of family 18 chitinases [103–105]. There is therefore a great potential for CHOS as an anti-inflammatory drug in patients with asthma. For a more detailed description of this and related topics, see the review by Muzzarelli in this special issue of Marine Drugs [143].

### 7.3. Increased bone strength

Mesenchymal stem cells from the bone marrow are able to differentiate into chondrocytes (cartilage), adipocytes (fat) and osteoblasts (bone). Osteoblasts produce osteoid; which is further mineralized to produce the bone matrix. Bone-tissue is mainly composed of bone matrix and osteoblasts. Chitosan and CHOS are known to increase the differentiation of mesenchymal stem cells to osteoblasts and to consequently facilitate the formation of bone-tissue [21,22].

The mineralization process and bone strength are dependent on Ca^2+^, which helps to support the structure. There is evidence that CHOS increase calcium-deposition in bone [22,144,145]. Jung *et al.* [144] found that CHOS could efficiently inhibit the formation of insoluble calcium-phosphate salts and consequently increase Ca^2+^ bioavailability and bone strength. They also found that CHOS (<5 kDa) gave increased calcium retention and decreased bone turnover in a rat osteoporosis model. This indicates that CHOS may have beneficial effects as a calcium fortifier in conditions of Ca^2+^ deficiency, such as in osteoporosis.

### 7.4. CHOS in gene therapy

Chitosans have been successfully used as vectors for delivery of genes (gene therapy) since the first report about 15 years ago [146,147]. Chitosan forms stable complexes with plasmid DNA and can be used as a vector for the administration of genes to mucosal tissues such as the lungs [148] and intestinal epithelium [149,150]. There are, however, certain drawbacks connected to the use of high molecular weight chitosans because of the low solubility at physiological pH, the high viscosity and the fact that the chitosan complexes often tend to form aggregates. By using CHOS instead of chitosan these drawbacks may be overcome [16]. Köping-Höggård *et al.* [16] showed that fully deacetylated CHOS (DP 24) formed stable complexes with plasmid DNA, and *in vitro* and *in vivo* experiments proved that these CHOS were effective vectors for delivery of genes [16,17]. It has been speculated that a delicate balance between the stability of the CHOS-DNA-complexes at lower pH-values (around pH 6) and their instability at higher pH-values (above pH 7) could be the reason for their efficiency [151]. This has recently been confirmed in detailed studies of how chitosan chemistry can be used to create an optimal balance between the stability of the complexes and their unpacking [152].

### 7.5. Prevention of bacterial adhesion to human cells

Some pathogens can initiate disease in humans by using carbohydrate binding proteins (lectins) to attach to complementary membrane-bound oligosaccharides on host cells [153,154]. Observed antibacterial and anti-infective effects of CHOS [13] are thought to be due to their ability to bind to the lectins on human pathogens and, consequently, prevent adhesion to human cells. *A priori*, one would expect the sequence of GlcNAc and GlcN units in CHOS to be important for binding affinity, and for ensuring selectivity for pathogens (*i.e.*, the CHOS should preferably not bind to lectins of the normal bacterial flora). In a recent study, it was shown that a mixture of 97% deacetylated tetramers significantly inhibited adhesion of certain enteropathogenic *Escherichia coli* strains to human colon adenocarcinoma epithelial (HT29) cells in tissue culture, whereas the binding of other *E. coli* strains was not inhibited [13]. Since pathogens vary in terms of their lectins, identification of both the target-lectins and the possible complementary CHOS are important when pursuing this application route of CHOS.

### 7.6. CHOS as a chitinase-inhibitor for preventing malaria

Malaria is caused by several species of the parasite *Plasmodium. P. falciparum* causes the most serious forms of malaria in humans, whereas *P. vivax, P. ovale* and *P. malarie* give a milder disease that is not generally fatal [155]. Each year, 350–500 million cases of malaria occur worldwide, and over one million people die, most of them young children in sub-Saharan Africa. There is no vaccine available, and the identification of molecular targets for vaccine development is of great importance. *Anopheles* mosquitoes transmit the malaria-parasite from one infected person to another and the most important control strategy for malaria today is to interfere with different stages in the life cycle of the *Plasmodium* parasite.

During its life cycle, the *Plasmodium* parasite must be capable of penetrating the chitin-containing periotrophic matrix surrounding the mosquito midgut, to make the mosquito infective. To do so, *Plasmodium* species secrete family 18 chitinases capable of degrading the periotrophic matrix [23,156–160]. The *P. falciparum* chitinase (PfCHT1), the *P. vivax* chitinase (PvCHT1) and the *P. gallinaceum* chitinase (PgCHT1) have been characterized [161–163]. *P. gallinaceum* is the only malaria parasite species where more than one chitinase gene has been identified, and PgCHT1 and PgCHT2 are both family 18 chitinases. One approach to vector control might be inhibition of the secreted *Plasmodium* chitinases by chitinase inhibitors that are taken up by the mosquito via the blood meal [23,156–160]. Several studies have convincingly shown that inhibition of *Plasmodium* chitinases indeed reduces infectivity [23,163,164]. CHOS may perhaps be developed as nontoxic, inexpensive small-molecule inhibitors of these chitinases.

### 7.7. Applications of chitosan/CHOS in wound-dressings

The use of chitosan in wound dressings has been explored to a certain extent and positive effects have been documented in several studies [14,165–169]. Similar positive effects have been documented for CHOS, which were shown to accelerate the wound healing process [14,15]. It is quite likely that chitosan is converted to CHOS by naturally occurring enzymes and that the activity observed for chitosan might in fact be caused by CHOS. It may thus be advantageous to use CHOS in wound dressings to get a more immediate effect.

CHOS are thought to accelerate wound healing by enhancing the functions of inflammatory and repairing cells [170–172]. For example, it has been shown that subcutaneous injection of hexamers of CHOS enhanced migration of polymorphonuclear cells in dogs [171]. Hexamers of *N-*acetylated and fully deacetylated CHOS were shown to induce persistent release of IL-8, a potent activator and chemoattractant of polymorphonuclear cells, from fibroblasts from rats *in vitro* [170]. It must be noted that most authors ascribe the beneficial effect of longer CHOS and (polymeric) chitosan on wound healing to the ability of these compounds to form biocompatible ordered tissue-like structures (see [167] and references therein).

Hemostatic effects may also contribute to the beneficial effects of chitosan/CHOS in wound dressings. Chitosan enhances platelet adhesion and aggregation [165,173] and increases the release of the platelet derived growth factor-AB (PDGF-AB) and the transforming growth factor-β1 (TGF-β1) from platelets in canine blood [173]. These two factors retract inflammatory cells which are thought to be important in wound healing. Chitosan also has the ability of causing erythrocytes to aggregate [169].

Minagawa *et al.* [166] compared wound break strength and the increase in collagenase activity in wounds in rats after exposure to monomers, oligomers and polymers of the chitin-group (GlcNAc/*N-*acetylated CHOS/chitin) and the chitosan-group (GlcN/fully deacetylated CHOS/chitosan). They found that all six compounds increased both the wound break strength and the collagenase activity. Overall, the non-acetylated compounds were most effective compared to the corresponding *N-*acetylated compounds. The oligomers of fully deacetylated CHOS were most effective for wound break strength and GlcN gave the highest activity of collagenase. The enzyme collagenase is produced mainly by fibroblasts and inflammatory cells and its activity is related to remodeling in wound healing [174].

### 7.8. Antifungal effects

The antifungal activity of chitosan was discovered already in 1979 [175], and has been utilized to inhibit fungal growth in crops [176]. The antifungal potential of CHOS has to a lesser extent been investigated. In 1984 Kendra and Hadwiger [177] tested the antifungal activity of monomers– heptamers of deacetylated CHOS on *Fusarium solani*, which is infectious to pea crops, and found that the heptamer was most effective.

Subsequent research has revealed that longer oligomers of CHOS (also called low molecular weight chitosan, LMWC) are more effective. LMWC (4.6 kDa, average DP of 23) shows antifungal activity against *Candida krusei* and inhibits spore germination in *Fusarium oxysporum* [178]. Seyfarth *et al.* [26] found antifungal effects of LMWC on different *Candida* species. Both DP and F_A_ of the chitosan/CHOS are of great importance for the antifungal potential and LMWC with low F_A_ so far seems to be the most promising type of compound [179].

The anti-fungal effect of LMWC seems to be caused by its interaction with lipids in the plasma membrane, leading to morphological changes and cell surface disruptions [180,181]. The composition of the fungal plasma-membrane seems to be important for the sensitivity against chitosan, and a higher content of polyunsaturated fatty acids makes the fungi more sensitive [182].

From literature studies, as well as from own unpublished work on non-medical use of LMWC as anti-fungals, it is clear that LMWC indeed have a considerable potential in this area. This is a good reason for giving the application of LMWC, to combat fungal infections in humans, more research attention than it has received so far.

## 8. Concluding Remarks and Future Perspectives

Despite major progress in the past decade, the production of pure CHOS with defined DP, F_A_ and P_A_ is still a challenge. However, it is now fully possible to carry out controlled and reasonably well understood enzymatic production processes that yield CHOS preparations that are enriched for certain known compounds. The outcome of such processes can be controlled by controlling the enzyme, the starting chitosan (primarily F_A_), and the extent to which the degradation reaction is allowed to develop. Further refinement of the production step may be achieved by using engineered enzymes with changed binding preferences in one or more of their subsites and by carrying out specific deacetylation steps with chitin deacetylases.

Techniques for further purification of CHOS as well as for sequence determination are now available, but are still quite challenging to exploit. Scaling up purification methods at an economically acceptable cost is another challenge, meaning that, from an economical point of view, it is probably cheaper to produce CHOS mixtures that are enriched for a bioactivity, rather than producing truly pure compounds. It is conceivable that the further development of CHOS as bioactive molecules may include further functionalization by chemical methods, for example by coupling groups to the reducing end.

These improved methods for producing (almost) pure, well-characterized CHOS will help to create a knowledge base for understanding how CHOS exert bioactivities. For example, it may soon be possible to determine the crystal structures of chitinases in complex with a CHOS acting as an inhibitor. Likewise, the interaction of CHOS with AMCase [183] or HCgp39 [184] may be assessed by structural studies.

CHOS have a remarkably wide spectrum of possible bioactivities. While highly promising, there is no doubt that these bioactivities need to be substantiated by further studies with well-defined CHOS preparations, as well as by fundamental research on the molecular mechanism behind the activity. Only then the great promise of converting an abundant bioresource, chitin, to CHOS-based medicines can be met.

## Figures and Tables

**Table 1:** Some properties of the five enzymes that are specifically discussed in this review.

Enzyme	GH fam	Extra CBM^1^	Mechanism	Endo/Exo	Processivity	Subsite specificity		
						−2	−1	+1
***Chitinases:***								
**ChiA**	18	Yes (1)	Retaining	Endo/exo^2^	Yes	A/D	A	A/D
**ChiB**	18	Yes (1)	Retaining	Endo/exo^2^	Yes	A/D	A	A/D
**ChiC**	18	Yes (2)	Retaining	Endo	No	A/D	A	A/D
**ChiG**	19	No	Inverting	Endo	No	A	A/D	A
***Chitosanase:***								
**Csn88**	46	No	Inverting	Endo	No	D/A	D/A	D/A

1 ChiA and ChiB are compact two domain enzymes containing an Fn3 domain and a chitin-binding domain classified as CBM5 in addition to their catalytic domain, respectively (Figures 1 and 2). The crystal structures of complete ChiA and ChiB are known (Figure 1). In ChiC, the catalytic domain is connected to an Fn3-like domain and a chitin-binding domain classified as CBM12 by a proline- and glycine-rich linker, which tends to be proteolytically cleaved *in vivo*. It has so far not been possible to determine the crystal structure of intact ChiC.

2 It has been shown that ChiA and ChiB primarily act as endo-processive enzymes on chitosan [118]. This is probably also the case on chitin, although there may be more exo-activity in this case [119]. In any case, the endo-/exo- difference is of little relevance for enzymes that act processively.

**Table 2:** Composition of dimer, trimer and tetramer fractions at different α-values during degradation of chitosan (F_A_ = 0.65) by ChiA, B and C. Data from Horn *et al.* [56]. Reproduced with permission from Wiley-Blackwell.

Enzyme	α	Dimer	Trimer	Tetramer
**ChiA**	0.15	81% AA 19% DA	81% DAA 19% ADA	100% -AA
	0.35	64% AA 36% DA	51% DAA 28% ADA 21% DDA	56% -AA 44% -DA
**ChiB**	0.11	86% AA 14% DA	71% DDA 29% AAA	100% -AA
	0.38	66% AA 34% DA	95% DAA 3% DDA 2% ADA	75% -AA 25% -DA
**ChiC**	0.20	100% AA	66% DAA 34% AAA	100% -AA
	0.38	81% AA 19% DA	100% DAA	100% -AA
